# Long-term peritoneal dialysis followed by kidney transplantation for Takayasu arteritis: a case report

**DOI:** 10.1186/s12882-019-1302-5

**Published:** 2019-04-18

**Authors:** Akane Yanai, Kiyotaka Uchiyama, Yoshitaka Ishibashi

**Affiliations:** 1 Division of Nephrology, Japanese Red Cross Medical Center, 4-1-22 Hiroo, Shibuya-ku, Tokyo, 150-8935 Japan; 2 Division of Endocrinology, Metabolism and Nephrology, Department of Internal Medicine, Keio University School of Medicine, 35 Shinanomachi, Shinjuku-ku, Tokyo, 160-8582 Japan

**Keywords:** Takayasu arteritis, End-stage renal disease, Peritoneal dialysis, Kidney transplantation, Internal iliac artery, Case report

## Abstract

**Background:**

Takayasu arteritis (TA) is a chronic vasculitis of unknown etiology that primarily affects large vessels. Although renal involvement is frequent in TA, patients with TA undergoing renal replacement therapy, especially long-term peritoneal dialysis (PD) and kidney transplantation (KTx), are rarely reported. We herein present the case of an elderly patient with TA treated by PD for more than 5 years and underwent KTx thereafter.

**Case presentation:**

A 69-year-old female diagnosed with TA at the age of 19 was treated by PD for seven and a half years for end-stage renal disease due to TA. Dialysate-to-plasma ratio of creatinine, which was well maintained during this period, reflected the efficacy of long-term PD. However, her residual renal function declined; she developed malnutrition, inflammation, and atherosclerosis syndrome and underwent living-related KTx from her husband. Due to the total occlusion of the external iliac arteries with compensatory development of the internal iliac arteries, the right internal iliac artery was used as the anastomosis site. After KTx, the patient developed chronic active antibody-mediated rejection; however, the graft function was maintained throughout the follow-up period. Despite severe aortic calcification and intermittent claudication in the legs, her condition did not worsen, and the blood flow of the graft was preserved.

**Conclusions:**

The current case illustrating the success of long-term PD and living-related KTx in maintaining kidney function in an elderly patient with TA is the first to demonstrate the potential of PD and KTx as feasible options for renal replacement therapy in TA accompanied by severe cardiac involvement.

## Background

Takayasu arteritis (TA) is a chronic vasculitis of unknown etiology that primarily involves the aorta and its primary branches [1]. Renal involvement in TA is frequent, primarily due to renal artery stenosis that leads to renovascular hypertension [2]. However, the number of patients who need renal replacement therapy is low because of the severe cardiovascular events that are often fatal. Among the renal replacement therapy options for patients with TA, hemodialysis is unsuitable because of the difficulty in creating arteriovenous fistulas as well as challenges in monitoring blood pressure and assessing blood flow to major organs. Conversely, reports documenting the clinical experience with peritoneal dialysis (PD) and kidney transplantation (KTx), which are likely preferable choices in patients with TA, have been limited.

We herein describe a patient with TA and end-stage renal disease who was treated by PD for more than 5 years before undergoing successful KTx.

## Case presentation

A 69-year-old female was diagnosed with TA at the age of 19 years, and steroid therapy controlled the diseases; however, her renal function declined gradually with renovascular hypertension developing as a complication of TA. At the age of 61, PD was initiated because hemodialysis was inappropriate because of severe aortic regurgitation and peripheral arteriosteogenesis that precluded the creation of an arteriovenous fistula.

**Fig. 1:**
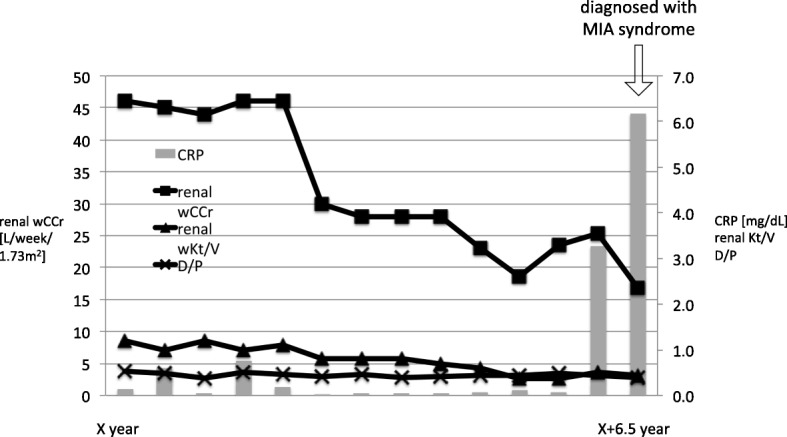
Clinical course after peritoneal dialysis initiation. Serum levels of C-reactive protein (CRP) gradually increased with a decreasing residual renal function (RRF), which led to the diagnosis of malnutrition, inflammation, and atherosclerosis (MIA) syndrome. Dialysate-to-plasma ratio of creatinine (D/P) was preserved throughout the clinical course of the patient. Abbreviations: wCCr, weekly creatinine clearance

The patient remained stable on PD for more than 5 years, with well-preserved residual renal function (RRF) and peritoneal function and only one episode of peritonitis, without any cardiocerebrovascular events. However, a gradual decline in RRF was accompanied by a concomitant increase in serum C-reactive protein (CRP) levels. The patient was evaluated for other inflammatory diseases, such as malignancies, infections, and autoimmune diseases including TA reactivation; however, nothing abnormal was detected. She also suffered from fatigue and vomiting; therefore, the increase in CRP indicating inflammation was considered to be due to malnutrition, inflammation, and atherosclerosis (MIA) syndrome resulting from inadequate renal replacement therapy with PD, evident from the RRF decline, leading to the accumulation of uremic solutes. Transition to hemodialysis or combination therapy with hemodialysis and PD were considered to improve her clinical condition; however, hemodialysis was challenging to the limitations associated with TA. Seven and a half years after the initiation of PD, she received a living-related KTx from her husband (Fig. 1).

**Fig. 2:**
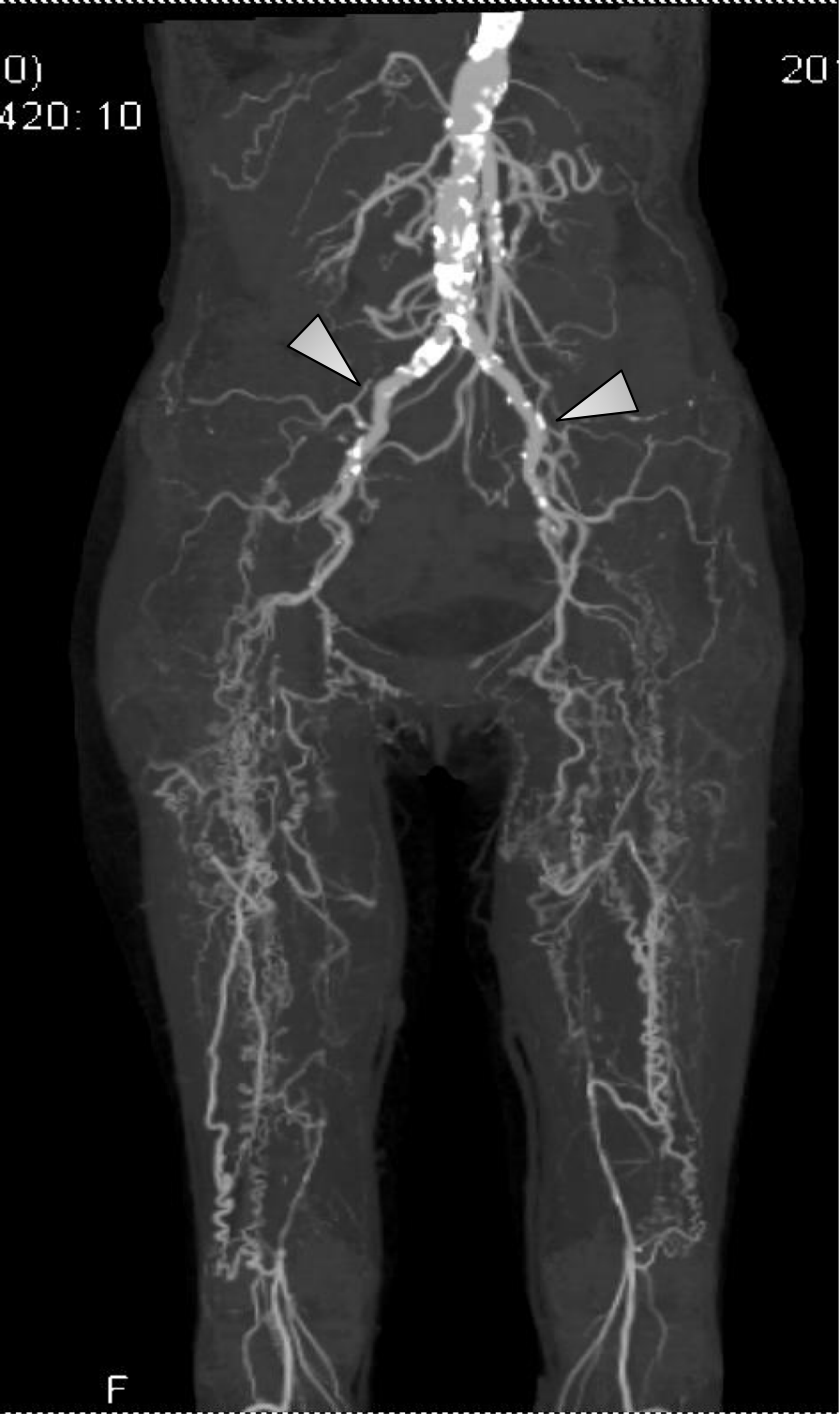
Takayasu arteritis involving aorta with skipped segments. Magnetic resonance angiography showing disruption of the bilateral external iliac arteries (arrow) and many corkscrew vessels extending from the internal iliac arteries toward the femurs. Internal iliac arteries are comparatively smooth

**Fig. 3:**
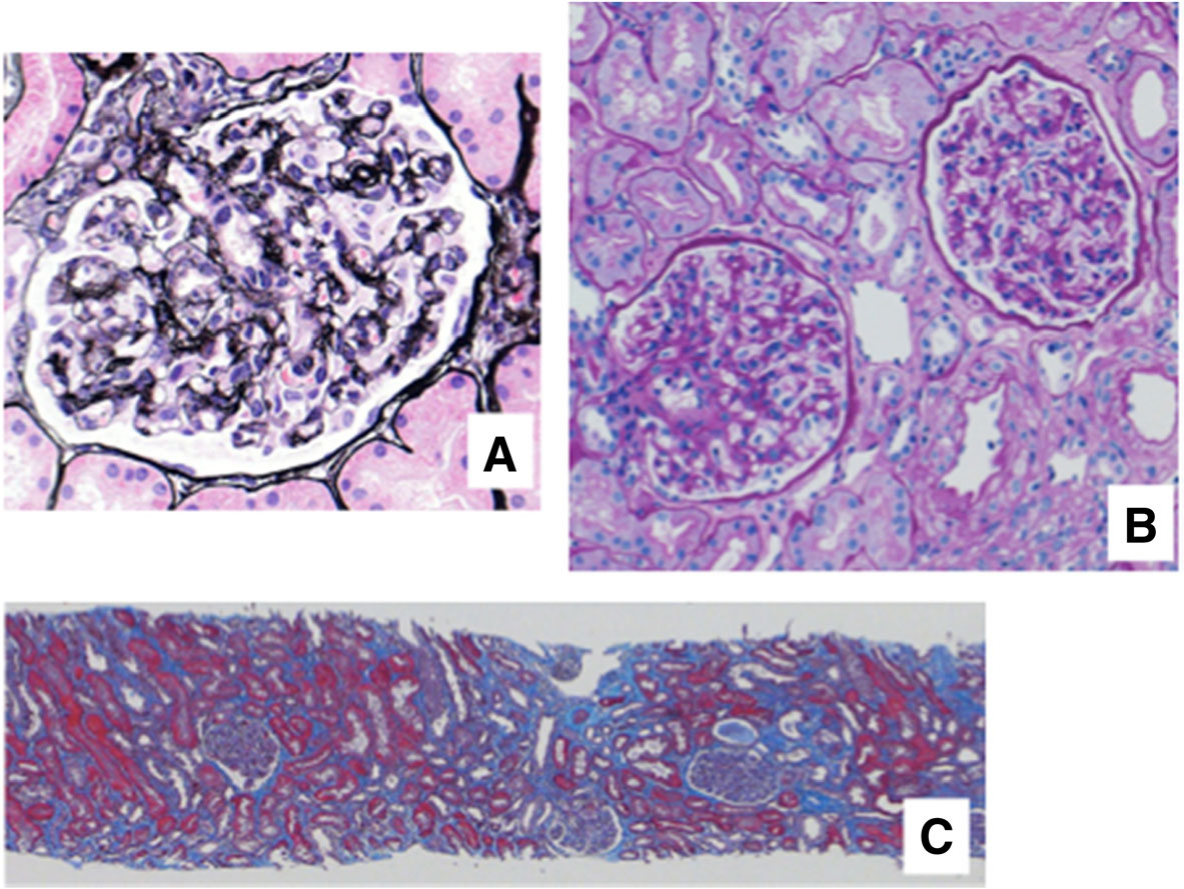
Histological evaluation of the biopsy specimen at 3 months after transplantation. The cortex-to-medulla and global sclerosis-to-glomeruli ratios were 7/3 and 2/16, respectively, indicating chronic active antibody-mediated rejection, mild toxic tubulopathy, moderate arteriosclerosis, and mild arterior hyalinosis. Banff scores were i0, t0, g2, ptc0, v0, ci0, ct0, cg1b, cv0, ah1, and aah0. Peritubular capillaries exhibit focal C4d positivity by immunofluorescence (C4d2). Simian virus 40 was negative by immunohistochemistry using the streptavidin-biotin method. **a** Periodic acid–methenamine–silver stain, × 400; (**b**) periodic acid–Schiff stain, × 200; (**c**) Masson trichrome stain, × 40

**Fig. 4:**
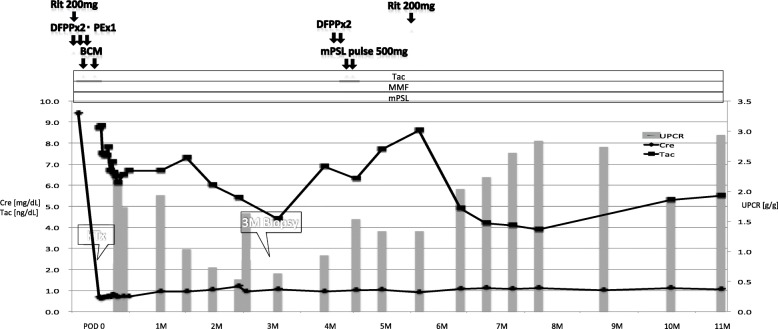
Clinical course after kidney transplantation. Immunosuppression was induced with tacrolimus (Tac), mycophenolate mofetil (MMF), methylprednisolone (mPSL), and basiliximab (BCM). Rituximab (Rit), two sessions of double filtration plasmapheresis (DFPP), and one session of plasma exchange were also induced as desensitization therapy because of ABO incompatibility and donor-specific antibodies positivity. The renal biopsy at 3 months revealed chronic active antibody-mediated rejection, and the patient was treated with pulse mPSL, Rit, and two sessions of DFPP. The urinary protein remained positive; however, the patient preserved renal graft function throughout the follow-up period. Abbreviations: Cre, creatinine; KTx, kidney transplantation; M, months; PE, plasma exchange; UPCR, urinary protein-to-creatinine ratio

The immunological profile of the KTx was as follows: ABO, incompatible (from B+ to O+); human leukocyte antigen (HLA), six mismatches; complement-dependent cytotoxicity, negative; flow cytometric lymphocyte crossmatch test, negative; and donor-specific antibodies (DSA), positive against HLA-DQ4 with a mean fluorescence intensity of 3683. She had severe arterial calcifications: bilateral external iliac arteries were disrupted, and many corkscrew vessels ran from the internal iliac arteries toward the femurs as compensatory vessels (Fig. 2). The internal iliac arteries were comparatively smooth, and living KTx surgery using the left donor kidney was performed in her right iliac fossa by end-to-side anastomosis with the right internal iliac artery. The intermittent claudication in the legs did not worsen after the surgery. The right ankle brachial index declined from 0.40 to 0.31, which was considered negligible, because a change of 0.15 or above is considered significant [3]. Total ischemic time, warm ischemic time, and onset of diuresis were 1 h 18 min 9 s, 5 min 16 s, and 14 min 53 s, respectively. Immunosuppression was induced with tacrolimus, mycophenolate mofetil, methylprednisolone, and basiliximab. Rituximab, two sessions of double-filtration plasmapheresis (DFPP), and one session of plasma exchange were also performed as desensitization therapy because of ABO incompatibility and DSA positivity. Her creatinine level was almost stable; however, urinary protein was detectable even 3 months later. The protocol and episode biopsy performed at the time revealed chronic active antibody-mediated rejection (CAAMR), with Banff 2015 [4] scores of g2 and cg1b (Fig. 3). Peritubular capillaries exhibited focal C4d positivity by immunofluorescence (C4d2). We considered that this histological change was caused by anti-HLA antibodies and was not due to ABO incompatibility because ABO-related AMR tends to occur especially within 2–7 days posttransplant and does not occur more than 1 month posttransplant owing to accommodation [5, 6], which leads to the excellent outcome in ABO-incompatible recipients in our country [7]. The patient was treated with pulse methylprednisolone, rituximab, and two sessions of DFPP for CAAMR. Although the urinary protein was still positive for 1 year after KTx, the mean fluorescence intensity of DSA against HLA-DQ4 declined to 2426 and the renal graft function was preserved throughout the follow-up period (Fig. 4).

## Discussion and conclusions

TA is common in Asia, with a prevalence of more than 0.004% in Japan (the absolute number is more than 6000) [8]. Although end-stage renal disease is one of the major complications of TA, hemodialysis induction can be difficult because of several factors including insufficient arterial flow for vascular access, which may cause distal ischemia and subclavian steal syndrome, difficulty in determining blood pressure, and hemodynamic instability during hemodialysis because of TA-associated cardiovascular complications. In the absence of vascular access, PD can be an optimal treatment strategy [9, 10]. A previous study reported the nearly complete preservation of the morphology of the peritoneal membrane, which might be expected because TA primarily affects large vessels, leading to relatively maintained peritoneal function [11]. In the present case, the peritoneum was found to be nearly normal based on peritoneal biopsy findings at the time of the first episode of peritonitis, which occurred approximately 6 years after the PD initiation. The dialysate-to-plasma ratio of creatinine was also preserved, reflecting the efficacy of long-term PD in the current patient (Fig. 1).

To date, there is only one report of a young female patient with TA who underwent KTx [12]. Iliac arteries are not major regions of aortic involvement in TA (13.3–15.8%) [13–15]. In the current case, although the external iliac arteries were completely disrupted, the blood flow of the lower extremities was not affected and that of the graft was also preserved throughout the follow-up period. The patient developed CAAMR but preserved renal graft function. Additional studies are necessary to assess long-term graft prognosis, incidence of cardiovascular events, and survival after KTx in patients with TA.

In conclusion, the current case illustrates the success of long-term PD and subsequent living-related KTx in maintaining kidney function in an elderly patient with TA. This is the first report to demonstrate the potential of PD and KTx as feasible options for renal replacement therapy in TA accompanied by severe cardiac involvement.

## Abbreviations

CAAMR: Chronic active antibody-mediated rejection
CRP: C-reactive protein
DFPP: Double-filtration plasmapheresis
DSA: Donor-specific antibodies
HLA: Human leukocyte antigen
KTx: Kidney transplantation
MIA: Malnutrition, inflammation, and atherosclerosis
PD: Peritoneal dialysis
RRF: Residual renal function
TA: Takayasu arteritis
